# Diagnostic Accuracy of Next Generation Sequencing Panel using Circulating Tumor DNA in Patients with Advanced Non-Small Cell Lung Cancer: A Systematic Review and Meta-Analysis

**DOI:** 10.36469/jheor.2020.17088

**Published:** 2020-09-14

**Authors:** Mariana M. Sebastião, Rodrigo S. Ho, João Paulo V. de Carvalho, Micha Nussbaum

**Affiliations:** 1 Roche Diagnostics, São Paulo, Brazil; 2 Roche Diagnostics LATAM, São Paulo, Brazil

**Keywords:** Meta-analysis, circulating tumor DNA, next-generation sequencing, non-small cell lung cancer

## Abstract

**Background/Objectives:**

Until now, no meta-analysis has been published to evaluate the diagnostic performance of next-generation sequencing (NGS) panel using circulating tumor (ctDNA) in patients with advanced non-small cell lung cancer (aNSCLC). The aim of the study was to carry out a systematic review and a meta-analysis in order to determine the accuracy of NGS of ctDNA to detect six oncogenic driver alterations: epidermal growth factor receptor (EGFR); anaplastic lymphoma kinase (ALK); ROS proto-oncogene 1, receptor tyrosine kinase (ROS-1); serine/threonine-protein kinase B-RAF (BRAF); RET proto-oncogene (RET); and MET proto-oncogene, receptor tyrosine kinase (MET) exon 14 in patients with aNSCLC.

**Methods:**

MEDLINE/PubMed, Cochrane Library, Latin American and Caribbean Health Sciences Literature (LILACS), and Centre for Reviews and Dissemination databases and articles obtained from other sources were searched for relevant studies that evaluate the accuracy (sensitivity and specificity) of NGS using ctDNA in patients with aNSCLC. The studies were eligible when NGS of ctDNA was compared with tissue tests to detect at least one of the six oncogenic driver alterations. Diagnostic measures (sensitivity and specificity) were pooled with a bivariate diagnostic random effect. All statistical analyses were performed with software R, v.4.0.0.

**Results:**

Ten studies were eligible for data extraction. The overall pooled estimates of sensitivity and specificity were 0.766 (95% CI: 0.678–0.835); 0.999 (95% CI: 0.990–1.000), respectively.

**Conclusions:**

The analysis has demonstrated that the NGS panel using ctDNA has a high accuracy to identify the six actionable oncogenic driver alterations in patients with aNSCLC. Therefore, it can be considered a reliable alternative to guide the patients with aNSCLC to the right treatment who cannot undergo an invasive procedure or have insufficient tissue material for molecular tests.

## INTRODUCTION

Lung cancer is the cancer with the greatest incidence all over the world (11.6% of all cases) and it also represents the main cause of cancer death.^1^,^2^ The majority of the patients with lung cancer are diagnosed in metastatic stage which has a 5-year survival rate of 4.7%.^3^ Among the histological types, non-small cell lung cancer (NSCLC) is the most common, representing around 80% to 85% of all cases in which approximately 40% are adenocarcinoma, 25% to 30% are squamous carcinoma, and 10% to 15% are large cell carcinomas.^4^–^6^

In the era of precision medicine, the therapeutic decisions for lung cancer are very dependent on histological and molecular characterization.^7^ NSCLC is considered a heterogeneous disease with diverse molecular characteristics.^8^ NSCLC has become an eminent example of precision medicine among solid tumors.^9^

In personalized medicine, patients are selected for a specific treatment based on the presence of specific biomarkers which indicates a greater chance of the patient to benefit from the treatment.^10^ Therapeutic options for NSCLCs have increased significantly with the emergence of targeted therapies and immunotherapies.^10^

The National Comprehensive Cancer Network (NCCN) guideline recommends that patients with aNSCLC should be tested for epidermal growth factor receptor (EGFR); anaplastic lymphoma kinase (ALK); ROS proto-oncogene 1, receptor tyrosine kinase (ROS-1); serine/threonine-protein kinase B-Raf (BRAF); MET proto-oncogene, receptor tyrosine kinase (MET) exon 14 skipping; RET proto-oncogene (RET); neurotrophic receptor tyrosine kinase (NTRK); and programmed death-ligand 1 (PD-L1).^11^ The NCCN guideline strongly advises the use of broad molecular profiling in order to identify rare driver mutations for which drugs may be available.^11^ However, approximately 20% to 30% of patients with NSCLC have insufficient tissue material to assess oncogenic driver mutations.^12^,^13^ In this situation, the NCCN guideline also recommends plasma testing in NSCLC patients in order to detect EGFR, ALK, ROS-1, BRAF, MET, and RET.^11^

Liquid biopsy is a less invasive procedure that can access the bloodstream through a needle stick, avoiding the risks of tissue biopsies. Circulating tumor DNA (ctDNA) can be used to provide the same genetic information as a tissue biopsy necessary to interrogate key companion diagnostics.^14^ Besides, liquid biopsy can also overcome other limitations of tissue biopsies such as detecting tumor heterogeneity and the molecular changes in cancer cells after they are exposed to therapy.^14^–^17^

The College of American Pathologists (CAP), the International Association for the Study of Lung Cancer (IASLC), and the Association for Molecular Pathology (AMP) recommends liquid biopsy not as a replacement for tissue biopsy but in cases that there is insufficient tumor tissue specimens or in cases where tissue specimens are not feasible.^18^ The CAP/IASLC/AMP considers next-generation sequencing panel using ctDNA (ctDNA NGS) a reliable platform in which it can assess single-base variants, indels, copy number changes, and translocations and it can reach acceptable sensitivity and optimal specificity.^18^

Until now, no meta-analysis has been published to evaluate the diagnostic performance of ctDNA NGS in patients with advanced NSCLC. Thus, we conducted a systematic review and a meta-analysis in order to investigate the diagnostic accuracy of ctDNA NGS in detecting the six oncogenic driver mutations: EGFR, ALK, ROS-1, BRAF, RET, and MET exon 14 in patients with advanced NSCLC.

## METHODS

### Study Design

A comprehensive electronic search was performed and included studies that were published until May 2019 in the following databases: MEDLINE/PubMed, The Cochrane Library, Latin American and Caribbean Health Sciences Literature (LILACS), and Centre for Reviews and Dissemination. A gray literature search was also performed in order to detect non-indexed publications.

### Search Strategy and Study Selection

Search strategy was defined in order to answer the following question: “Is the ctDNA NGS panel an accurate test to detect oncogenic driver mutations in patients with aNSCLC when compared to tissue genotyping method?” (Table S1).

Specific keywords and terms for each database were considered. The strategies used in each database are shown in Table S2. Article language was limited to English.

Studies were eligible when ctDNA NGS was applied to detect at least one of the following biomarkers in aNSCLC patients: EGFR, ALK, ROS-1, BRAF, RET, and MET exon 14 alterations. Also, studies must use any tissue genotyping method as the gold standard. Exclusion criteria included the absence of sensitivity or specificity data, the analysis of patients with diagnoses other than aNSCLC or healthy subjects. Two reviewers (Ho and Sebastião) evaluated eligibility of studies according to these criteria.

### Data Extraction

Two reviewers (Ho and Sebastião) extracted data from all eligible studies. Name of first author, year of publication, histologic type of NSCLC, clinical stage, comparator (“gold standard”), and diagnostic results for EGFR, ALK, ROS-1, BRAF, RET, and MET exon 14 alterations—true positive (TP), false positive (FP), false negative (FN), and true negative (TN)—were collected from eligible studies. EGFR T790M was also considered in our analysis. Genomic alterations in EGFR, ALK, ROS-1, BRAF, RET, and MET exon 14 evaluated by tissue genotyping were considered the “gold standard.”

### Quality Assessment

The quality of included studies was assessed using the standardized instrument Quality Assessment of Diagnostic Accuracy Tests (QUADAS-2). QUADAS-2 is designed to assess the quality of primary diagnostic accuracy studies. This tool evaluates the studies based on four key domains: patient selection, index test, reference standard, and flow and timing. Two reviewers (Ho and Sebastião) evaluated the quality of eligible studies.^19^,^20^

### Statistical Analysis

To assess the test accuracy, data of TP, FP, FN, and TN were tabulated and stratified by study. These diagnostic data were used to calculate the pooled sensitivity, specificity, and diagnostic odds ratio (DOR).

Statistical analysis was performed using the summary Receiver Operating Characteristic (sROC) and the bivariate approach. The sROC is the standard method for meta-analysis of diagnostic accuracy. The bivariate model jointly analyzes the sensitivity and specificity, considering any correlation between these two parameters using a random effect model.^21^

The heterogeneity between studies was measured by Cochran’s Q test to test the inconsistency index (I^2^) (p < 0.05 or I^2^ >50%).^22^

All statistical analysis was performed with software R, v.4.0.0. The bivariate was fitted by the mada package which is based on the bivariate model of Reitsma et al, bivariate random effects model.^21^

## RESULTS

### Characteristics of Eligible Studies

**Figure 1:**
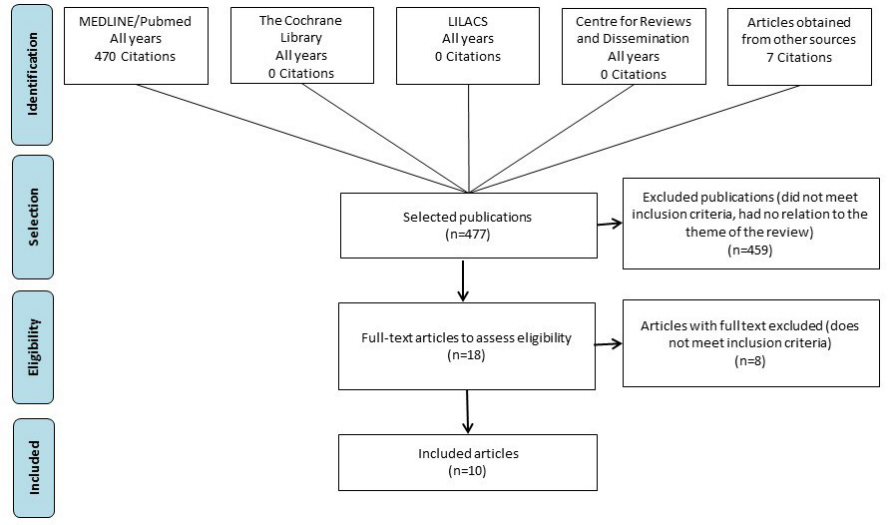
Study Selection Flowchart Abbreviations: LILACS, Latin American and Caribbean Health Sciences Literature.

Searches returned 477 citations that were published until May 2019. After screening using the predefined eligibility criteria, 10 studies were included (Figure 1 ).^13^,^23^–^31^

General characteristics of the 10 studies included in the review are reported on Table 1. A total of 2116 results from patients with histologically-confirmed diagnosis of advanced NSCLC with ctDNA NGS were evaluated for the six oncogenic driver mutations. Only data from advanced clinical stages were considered in the study. Two studies selected also reported data from patients with NSCLC in early (I–IIIA) stages, but only data from advanced (IIIB–IV) stages were considered. All studies evaluated the accuracy of NGS ctDNA with tissue genotyping, which may have included polymerase chain reaction (PCR) testing, fluorescence in situ hybridization (FISH) and/or immunohistochemical (IHC), or Sanger sequencing. Exclusion reasons for full-text excluded citations are described in Table S3.

### Quality of Eligible Studies

The methodologic quality of the studies was evaluated by QUADAS-2 and they are summarized in Table 2.

### Diagnostic Accuracy

**Figure 2:**
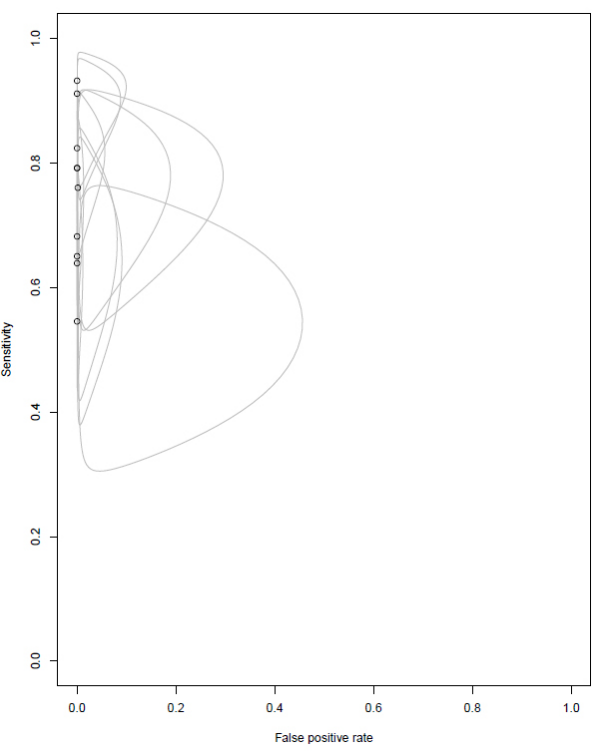
Confidence Interval of the Studies Included

The data extracted from each study regarding the six oncogenic driver mutations is described in Table 3. All the studies have demonstrated a specificity of 100%, except the Leighl et al^26^ that had one case of FP in MET exon 14. This alteration was not evaluated with other ctDNA methodologies as a reflex test. One of the studies, Rachilio et al,^24^ did show two FP results for EGFR mutations that were detected by NGS ctDNA, but considered EGFR wild type by tumor genotyping. Both EGFR mutations were confirmed by droplet digital PCR (ddPCR), so they were considered TP results. The sensitivity among the studies ranged from 54.5% to 92.1%. Figure 2 shows the plots of confidence regions of each study, describing the uncertainty of the pair of sensitivity and FP rate (1-specificity).

The pooled sensitivity and specificity of NGS ctDNA were 0.766 (95% CI: 0.678–0.835) and 0.999 (95% CI: 0.990–1.000), respectively. The diagnostic accuracy, area under curve, reached 0.99.

The pooled DOR, which is the general diagnostic test performance, was 616.5 (95% CI: 263.0–1445.0). Heterogeneity investigation was performed among included studies, but they were considered homogeneous (Cochrane’s Q *P* = 0.437 and I^2^=0%).

As nine of 10 studies included in the analysis had a specificity of 100% and one study had a specificity of 99.9%, the sROC curve could not be generated.

## DISCUSSION

The NCCN guideline recommends plasma testing to evaluate EGFR, ALK, ROS-1, BRAF, RET, and MET alterations when there is insufficient tissue material to guide the use of target therapies in patients with advanced or metastatic NSCLC.^11^ The results demonstrated that NGS ctDNA has a high accuracy to detect the six oncogenic driver mutations.

The meta-analysis demonstrated that NGS ctDNA reached an optimal specificity of 0.999 (95% CI: 0.990–1.000) which is a very important result to give confidence in prescribing target therapies in patients who will not be FPs for the six oncogenic driver mutations evaluated. The sensitivity reached an acceptable value of 0.766 (95% CI: 0.678–0.835).

These results support the recommendation by CAP/IASLC/AMP, which suggests that patients with positive results for EGFR, ALK, ROS-1, or BRAF with ctDNA NGS should start first-line therapy, as the results are considered reliable. However, a negative result from ctDNA NGS for oncogenic driver mutations cannot exclude therapies and further investigation is required.^18^ Therefore, the CAP/IASLC/AMP considers liquid biopsy not as a replacement for tissue biopsy but as an alternative when there is insufficient tumor tissue specimens or in cases where tissue specimens are not feasible.^18^ It is important to highlight that this recommendation was published before the FDA approval for MET exon 14 target therapy and RET fusion target therapy. With the MET exon 14 and RET fusion target therapies approvals in other countries, more guidelines may recommend the detection of these oncogenic driver mutations with tissue and plasma tests.

The comparator in systematic review was restricted to tissue genotyping in order to assess the sensitivity of the ctDNA NGS. However, the limitations of using tissue genotyping as the “gold standard” is the tumor heterogeneity which might be missed by tissue biopsies.^32^,^33^ Therefore, tumor heterogeneity can reduce overall concordance between plasma and tissue.^34^ Jiang et al. have shown that subjects with stage II–IV NSCLC showed more somatic mutations in plasma than tissue samples.^34^ One of the studies, Leighl et al,^26^ had a FP case in MET exon 14 that was detected by ctDNA NGS, but it was not detected by tissue genotyping. As this FP result was not evaluated with other ctDNA methodology as a reflex test, the MET exon 14 could be due to the heterogeneity of the tumor.

The present study demonstrated the feasibility of using ctDNA NGS in detecting six oncogenic driver mutations to help guide the target therapies in patients with aNSCLC. However, ctDNA NGS has also the potential to monitor patients’ response to therapies (target and immune therapies) and resistance mutations. Currently, the use of ctDNA is limited to cancer in advanced stages due to its low concentration in the early stages.

## CONCLUSION

In conclusion, our meta-analysis supports the use of ctDNA NGS in clinical practice for those patients with advanced NSCLC who cannot undergo an invasive procedure or have insufficient tissue material for molecular tests. This technology provides a reliable alternative to guide the patients to the right treatment according to their molecular characteristics.

## Supplementary Information



## Figures and Tables

**Table 1:** Characteristics of Eligible Studies

Study	Year	Comparator	Clinical Stage	Driver Mutation
Rachiglio et al^24^	2016	NGS tissue and confirmed with ddPCR in tissue and plasma	Advanced	EGFR
Liu et al^13^	2018	NGS tissue	Advanced^a^	EGFR
				ALK
				BRAF
				RET
Guibert et al^25^	2018	Tumor genotyping^b^ and ddPCR in plasma	Advanced	EGFR
				ALK
				ROS-1
				BRAF
				MET exon 14
Paweletz et al^30^	2016	Tissue genotype by FISH, PCR, or NGS	Advanced	EGFR
				ALK
				ROS-1
				RET
Wang et al^28^	2016	IHC and FISH	Advanced	ALK
Cui et al^23^	2017	IHC and FISH	Advanced^a^	ALK
Veldore et al^27^	2018	Real time PCR	Metastatic	EGFR
Leighl et al^26^	2019	Standard-of-care tissue test^c^	Advanced	EGFR
				ALK
				ROS-1
				BRAF
				MET exon 14
Pritchett et al^31^	2019	NGS tissue	Advanced	EGFR
				ALK
				ROS-1
				BRAF
				MET exon 14
Yao et al^29^	2017	NGS tissue	Advanced^a^	EGFR
				ALK
				RET

Abbreviations: ALK, anaplastic lymphoma kinase; BRAF, serine/threonine-protein kinase B-Raf; EGFR, epidermal growth factor receptor; FISH, fluorescence in situ hybridization; IHC, immunohistochemical; NGS, non-genetic sequencing; NTRK, neurotrophic receptor tyrosine kinase; MET, MET proto-oncogene, receptor tyrosine kinase; PCR, polymerase chain reaction; PD-L1, programmed death-ligand 1; RET, RET proto-oncogene; ROS-1, ROS proto-oncogene 1, receptor tyrosine kinase.

a These studies also included patients in early stages but only data from advanced stage were considered.

b Methods of tissue genotyping were not described.

c NGS, PCR “hotspot” testing, FISH and/or IHC, or Sanger sequencing.

**Table 2:** Included Articles Quality Assessment According to QUADAS-2

Study	Risk of Bias				Applicability Concerns		
	Patient Selection	Index Test	Reference Standard	Flow and Timing	Patient Selection	Index Test	Reference Standard
Rachiglio et al^24^	LR	?	?	LR	LR	?	?
Liu et al^13^	LR	?	?	LR	LR	LR	LR
Guibert et al^25^	LR	LR	LR	LR	LR	LR	LR
Paweletz et al^30^	LR	HR	LR	LR	LR	HR	LR
Wang et al^28^	LR	HR	LR	HR	LR	?	LR
Cui et al^23^	HR	HR	LR	LR	?	HR	LR
Veldore et al^27^	LR	LR	LR	?	LR	LR	LR
Leighl et al^26^	LR	?	LR	LR	LR	?	LR
Pritchett et al^31^	LR	LR	LR	HR	LR	LR	LR
Yao et al^29^	LR	?	?	LR	LR	?	?

Abbreviations: ?, unclear risk; HR, high risk; LR, low risk; QUADAS-2, Quality Assessment of Diagnostic Accuracy Tests.

**Table 3:** Data Extracted from Each Study Included (EGFR, ALK, ROS-1, BRAF, RET, and MET exon 14)

Study	TP	FN	FP	TN	Sensitivity (95% CI)	Specificity (95% CI)
Rachiglio et al^24^	19	5	0	20	0.792 (0.578–0.929)	1.000 (0.832–1.000)
Liu et al^13^	13	7	0	84	0.650 (0.408–0.846)	1.000 (0.957–1.000)
Guibert et al^25^	41	3	0	76	0.932 (0.813–0.986)	1.000 (0.953–1.000)
Paweletz et al^30^	42	9	0	141	0.824 (0.691–0.916)	1.000 (0.974–1.000)
Wang et al^28^	19	5	0	36	0.792 (0.578–0.929)	1.000 (0.903–1.000)
Cui et al^23^	12	10	0	10	0.545 (0.322–0.756)	1.000 (0.692–1.000)
Veldore et al^27^	41	4	0	87	0.911 (0.788–0.975)	1.000 (0.958–1.000)
Leighl et al^26^	38	12	1	689	0.760 (0.618–0.869)	0.999 (0.992–1.000)
Pritchett et al^31^	23	13	0	711	0.639 (0.462–0.792)	1.000 (0.995–1.000)
Yao et al^29^	15	7	0	95	0.682 (0.451–0.861)	1.000 (0.962–1.000)

Abbreviations: ALK, anaplastic lymphoma kinase; BRAF, serine/threonine-protein kinase B-Raf; EGFR, epidermal growth factor receptor; FN, false negative; FP, false positive; MET, MET proto-oncogene, receptor tyrosine kinase; RET, RET proto-oncogene; TP, true positive; TN, true negative.
