# Supplementary material for: Diagnostic Accuracy of Next Generation Sequencing Panel using Circulating Tumor DNA in Patients with Advanced Non-Small Cell Lung Cancer: A Systematic Review and Meta-Analysis
Source: J Health Econ Outcomes Res. 2020 Sep 14;7(2):158–63. doi: 10.36469/jheor.2020.17088 (PMC7539761; doi:10.36469/jheor.2020.17088)
Supplement: Supplementary file 1 [file jheor-7-2-17088-s01.pdf]

JHEOR

Journal of Health Economics and Outcomes Research

Supplementary Online Material

Sebastião MM, Ho RS, de Carvalho JPV, Nussbaum M.Diagnostic accuracy of next generation sequencing panel using circulating tumor DNA in patients with advanced non-small cell lung cancer: a systematic review and meta-analysis. *JHEOR*. 2020;7(2):158-163. doi:10.36469/jheor.2020.17088

Table S1. PICO question
Table S2. Search strategy
Table S3. Excluded studies

This supplementary material has been provided by the authors to give readers additional information about their work.

This is an open-access article distributed under the terms of the Creative Commons Attribution 4.0 International License (CCBY-4.0). View this license's legal deed at http://creativecommons.org/licenses/by/4.0 and legal code at http://creativecommons.org/licenses/by/4.0/legalcode for more information.

Table S1. PICO question

Acronym (definition)	Strategy
P (population)	Advanced Non-Small Cell Lung Cancer
I (intervention)	NGS panel on ctDNA samples
C (comparator)	Any tissue genotyping method
O (outcome)	Accuracy: sensitivity and specificity

Table S2. Search strategy

Database	Strategy
**Pubmed**	(“Carcinoma, Non-Small-Cell Lung”[Mesh] OR “Carcinoma, Non Small Cell Lung” OR “Carcinomas, Non-Small-Cell Lung” OR “Lung Carcinoma, Non-Small-Cell” OR “Lung Carcinomas, Non-Small-Cell” OR “Non-Small-Cell Lung Carcinomas” OR “Nonsmall Cell Lung Cancer” OR “Non-Small-Cell Lung Carcinoma” OR “Non Small Cell Lung Carcinoma” OR “Carcinoma, Non-Small Cell Lung” OR “Non-Small Cell Lung Cancer”) AND ((“next-generation sequencing”) OR (“next generation sequencing”))
**Lilacs**	(“Carcinoma, Non-Small-Cell Lung” OR “Carcinoma, Non-Small Cell Lung” OR “Non-Small Cell Lung Cancer” OR “Non-Small-Cell Lung Carcinoma”) AND (“next generation sequencing”)
**Cochrane Library**	(“Carcinoma Non-Small-Cell Lung”) AND (“next generation sequencing”)
**CRD**	(Carcinoma Non-Small-Cell Lung) AND (next generation sequencing)

Abbreviations: CRD: Centre for Reviews and Dissemination; LILACS: Latin American and Caribbean Health Sciences Literature

Table S3. Excluded studies

Author, year	Reasons for exclusion
Schwaederlé, 2017	Absence of specificity data
Couraud, 2014	Inclusion of patients with diagnosis other than aNSCLC
Reckamp, 2016	Inclusion of healthy subjects
Krug, 2018	Absence of specificity data
Newman, 2014	Inclusion of healthy subjects
Xu, 2017	Inclusion of patients with staging other than advanced
Malapelle, 2017	Inclusion of patients with diagnosis other than aNSCLC
Plagnol, 2018	Comparator other than a tissue test

REFERENCES

1. Malapelle U, Mayo de-Las-Casas C, Rocco D, Garzon M, Pisapia P, Jordana-Ariza N, et al. Development of a gene panel for next-generation sequencing of clinically relevant mutations in cell-free DNA from cancer patients. *Br J Cancer*. 2017;116(6):802–810.
2. Newman AM, Bratman SV, To J, Wynne JF, Eclov NCW, Modlin LA, et al. An ultrasensitive method for quantitating circulating tumor DNA with broad patient coverage. *Nat Med*. 2014;20(5):548–554.
3. Plagnol V, Woodhouse S, Howarth K, Lensing S, Smith M, Epstein M, et al. Analytical validation of a next generation sequencing liquid biopsy assay for high sensitivity broad molecular profiling. Galli A, editor. *PLoS One*. 2018;13(3):e0193802.
4. Couraud S, Vaca-Paniagua F, Villar S, Oliver J, Schuster T, Blanche H, et al. Noninvasive diagnosis of actionable mutations by deep sequencing of circulating free DNA in lung cancer from never-smokers: a proof-of-concept study from BioCAST/IFCT-1002. *Clin Cancer Res*. 2014;20(17):4613–4624.
5. Schwaederlé MC, Patel SP, Husain H, Ikeda M, Lanman RB, Banks KC, et al. Utility of genomic assessment of blood-derived circulating tumor DNA (ctDNA) in patients with advanced lung adenocarcinoma. *Clin Cancer Res*. 2017;23(17):5101–5111.
6. Reckamp KL, Melnikova VO, Karlovich C, Sequist LV, Camidge DR, Wakelee H, et al. A highly sensitive and quantitative test platform for detection of NSCLC EGFR mutations in urine and plasma. *J Thorac Oncol*. 2016;11(10):1690–1700.
7. Krug AK, Enderle D, Karlovich C, Priewasser T, Bentink S, Spiel A, et al. Improved EGFR mutation detection using combined exosomal RNA and circulating tumor DNA in NSCLC patient plasma. *Ann Oncol*. 2018;29(3):700–706.
8. Xu T, Kang X, You X, Dai L, Tian D, Yan W, et al. Cross-platform comparison of four leading technologies for detecting EGFR mutations in circulating tumor DNA from non-small cell lung carcinoma patient plasma. *Theranostics*. 2017;7(6):1437–1446.
